# Randomised Clinical Trial for Postoperative Complications after Ex-PRESS Implantation versus Trabeculectomy with 2-Year Follow-Up

**DOI:** 10.1038/s41598-018-34627-w

**Published:** 2018-11-01

**Authors:** Shogo Arimura, Seiji Miyake, Kentaro Iwasaki, Makoto Gozawa, Takehiro Matsumura, Yoshihiro Takamura, Masaru Inatani

**Affiliations:** Department of Ophthalmology, Faculty of Medical Science, University of Fukui, Yoshida, Japan

## Abstract

We compared complications between Ex-PRESS implantation and trabeculectomy for 2 years after surgery. Sixty-four open-angle glaucoma eyes were randomly assigned to treatment with trabeculectomy (n = 32) or Ex-PRESS implantation (n = 32). The primary outcomes were postoperative complications, including reduction of the endothelial cell density (ECD) of the cornea, cataract progression and the frequency of other late postoperative complications. The Ex-PRESS group had significantly greater reduction of postoperative corneal ECD than the trabeculectomy group did at 2 years after surgery (*P* = 0.026). Among the corneal areas measured using specular microscopy, the superior area, where the Ex-PRESS tube was inserted, had significantly more severe corneal ECD reduction than the inferior area after 2 years (−17.6% in superior area and −11.7% in inferior area, *P* = 0.04). More cataract progression occurred in the trabeculectomy group than in the Ex-PRESS group (*P* = 0.04). Twelve eyes (37.5%) in the trabeculectomy group and 4 eyes (12.5%) in the Ex-PRESS group underwent cataract surgery (*P* = 0.019). The total number of other postoperative complications between 3 months and 2 years was significantly higher in the trabeculectomy group than in the Ex-PRESS group (*P* = 0.02). Although Ex-PRESS implantation might be associated with an increased rate of corneal endothelial cell loss compared with trabeculectomy, it is beneficial for preventing cataract progression after filtering surgery.

## Introduction

Trabeculectomy is a common filtering surgery for glaucoma patients with medically uncontrollable intraocular pressure (IOP). However, complications often develop after the surgery^1,2^. Especially, early postoperative complications due to over-filtration include a flat/shallow anterior chamber, choroidal detachment, suprachoroidal haemorrhage and hypotensive maculopathy, which are often associated with visual disturbance after surgery^3–5^. Filtering surgery using the Ex-PRESS glaucoma filtration device (Alcon Laboratories, Fort Worth, TX, USA) is a surgical option to reduce the frequency of complications resulting from over-filtration because the device is believed to offer more stable filtration compared with limbal tissue excision during trabeculectomy^6,7^. Previously, our clinical trial evaluating complications following Ex-PRESS implantation versus trabeculectomy in medically uncontrolled open-angle glaucoma (OAG) eyes, called ‘Complications Postoperatively of Ex-PRESS versus Trabeculectomy Study (CPETS)’, showed that Ex-PRESS implantation reduced the frequency of some early postoperative complications, including hyphema and flare elevation in the anterior chamber, compared with trabeculectomy^8^. Another prospective study comparing Ex-PRESS implantation with trabeculectomy reported fewer early postoperative complications with Ex-PRESS implantation^9^.

Despite the advantage of fewer early postoperative complications following Ex-PRESS implantation, late postoperative complications after the two procedures have not been well investigated. Corneal decompensation is a serious late complication after filtering surgery^10–12^. Recently, a case report described the occurrence of corneal decompensation after Ex-PRESS implantation in an eye with exfoliation glaucoma^13^. Loss of corneal endothelial cells frequently occurs after tube implantation in the anterior chamber when using the Baerveldt glaucoma implant and the Ahmed glaucoma valve^2,14,15^. Additionally, cataract progression is a common late complication in trabeculectomy. The complication seems to be related to peripheral iridectomy during the surgery because laser and incisional peripheral iridectomies are also associated with cataract progression^16,17^. Cataract progression was also compared in CPETS because phakic OAG eyes were included in the study^8^. The present study reports a comparison of the postoperative complications over 2 years, including quantification of the reduction of corneal endothelial cell density (ECD) and cataract progression following the two procedures.

## Methods

The present study protocol has been described in detail elsewhere^8^. CPETS was approved by the Institutional Review Board of Fukui University Hospital, Fukui, Japan, and written informed consents were obtained from all the patients. The protocol conformed to the tenets of Declaration of Helsinki. Patients were recruited between August 30, 2012 and February 13, 2015 at Fukui University Hospital. Japanese patients over 20 years with primary OAG (POAG) or exfoliation glaucoma with IOP ≥ 18 mmHg were enrolled in the study. They had phakic eyes without prior intraocular surgery. Eligible eyes were randomly assigned to undergo trabeculectomy (the trabeculectomy group) or Ex-PRESS implantation (the Ex-PRESS group). Mitomycin-C (0.4 mg/mL) was applied for 4 minutes during the surgery in all the eyes. Follow-up visits for the clinical trial were at 3, 6, 12 and 24 months after the surgery. Each pre- and postoperative examination included measurements of the slit lamp biomicroscopy, IOP measurement with Goldmann applanation tonometry, best corrected visual acuity and corneal specular microscopy.

### Primary outcome measures

The primary outcomes were postoperative complications, which included postoperative percent reduction of the corneal ECD, the coefficient of variance (CV) and hexagonal cell appearance rate (6A) of corneal endothelial cells compared with preoperative corneal ECD, CV and 6A, nuclear cataract progression and the frequency of postoperative complications between 3 and 24 months. The change in corneal ECD, CV and 6A of corneal endothelial cells between preoperative and postoperative visits was quantified in five areas (central, superior, inferior, nasal, and temporal) photographed with a non-contact specular microscope (NSP-9900 II, Konan, Nishinomiya, Japan) by a single experienced examiner (S.A.). The patients who underwent cataract surgery or reoperation due to insufficient IOP reduction were excluded from the analysis of corneal ECD. Nuclear cataract progression was quantified using the Lens Opacification Classification System III (LOCS-III)^18^. The light scattering intensity of the lens was evaluated using an anterior eye segment analysis system (EAS-1000, Nidek, Gamagori, Japan). A camera unit was used to obtain a two-dimensional linear image of the cross-section of the anterior segment along 0° with a 200 W light source. The light scattering intensity (cct; the intensity of light scattering per pixel in each layer) of the images was analysed using ImageJ.

### Secondary outcome measures

The secondary outcomes were the dependency of the corneal ECD reduction on the five photographed areas after surgery within each group, the comparisons of IOP and visual acuity between the two groups.

### Statistical analysis

JMP version 10.0 (SAS Institute, Inc. Cary, NC, USA) was used for statistical analysis. Univariate analysis was performed with Wilcoxon’s non-parametrical test, the paired t-test with Bonferroni correction and the χ^2^ test. *P* values were considered statistically significant if less than 0.05. The sample size was found to provide 80% power to prove (at a one-sided level of 0.05) the superiority of a significant outcome between the trabeculectomy and Ex-PRESS groups for an effect size of 0.7.

### Trial registration

Medical Information Network Clinical Trials Registry of Japan (identifier University Hospital Medical Information Network 000008680; date of access and registration, August 15, 2012).

## Results

### Patient recruitment

As shown in our previous report about early postoperative complications in the CPET study^8^, 32 eyes in the trabeculectomy group and 32 eyes in the Ex-PRESS group were postoperatively analysed. Table 1 summarises the characteristics of the patients in the study. There were no significant differences in baseline characteristics between the trabeculectomy and Ex-PRESS groups. The 1-year follow-up evaluation was completed for 30 eyes (93.8%) in the trabeculectomy group and 30 eyes (93.8%) in the Ex-PRESS group (*P* = 1.0), whereas the 2-year follow-up evaluation was completed for 25 eyes (78.1%) in the trabeculectomy group and 28 eyes (87.5%) in the Ex-PRESS group (*P* = 0.3).

**Table 1:** Demographic characteristics.

Characteristic	Trabeculectomy	Ex-PRESS	*P* value
	n = 32	n = 32	
Age, y	72.7 ± 9.6	70.7 ± 11.4	0.47*
Sex, Male/Female	13/19	14/18	0.80^†^
Side of surgical eye, Right/Left	17/15	14/18	0.45^†^
**Type**			
Primary open-angle glaucoma/Exfoliation glaucoma	19/13	20/12	0.80^†^
Number of preoperative medications	2.7 ± 0.7	2.6 ± 0.7	0.27*
Anterior chamber depth, mm	2.93 ± 0.45	2.94 ± 0.45	0.94*
Preoperative intraocular pressure, mm Hg	27.9 ± 10.7	27.2 ± 8.5	0.94*
Preoperative visual acuity, logMAR	0.33 ± 0.48	0.40 ± 0.43	0.28*
Axial length, mm	23.6 ± 1.6	24.2 ± 1.5	0.13*
Corneal thickness, mm	0.51 ± 0.05	0.51 ± 0.04	0.95*
**Automated perimetry programme 24-2**			
Eyes, No. (%)	11 (34)	16 (50)	0.75*
MD, mean (dB)	−10.2 ± 6.9	−11.3 ± 5.5	
**Automated perimetry programme 10-2**			
Eyes, No. (%)	18 (56)	13 (41)	
MD, mean (dB)	−22.0 ± 7.6	−22.0 ± 9.0	0.81*
Fixation loss			
No. (%)	3 (9)	3 (9)	1.00

LogMAR = logarithm of the minimal angle of resolution, MD = mean deviation.

*Mann–Whitney nonparametric test (data are mean values ± standard deviation).

^†^χ^2^ rank test.

### Primary outcomes

#### Corneal ECD after surgery

The corneal ECD (average of the five areas) was 2,487 ± 275 cell/mm^2^ in the trabeculectomy group and 2,564 ± 329 cell/mm^2^ in the Ex-PRESS group before surgery. The Ex-PRESS group had a significantly greater percent reduction in the postoperative corneal ECD than the trabeculectomy group did at 6, 12, and 24 months after surgery (Table 2) (P = 0.046 at 6 months; *P* = 0.004 at 12 months; and *P* = 0.026 at 24 months). The CV of corneal endothelial cell calculated by the average of the five areas was 0.369 ± 0.061 and 0.370 ± 0.072 in the trabeculectomy and Ex-PRESS groups, respectively, before surgery (*P* = 0.70). The postoperative percent reduction of CV during the follow-up period also exhibited no significant differences between the two groups. (0.03% ± 17.7% vs 2.60% ± 14.6%, *P* = 0.49 at 3 months; 1.87% ± 15.5% vs 2.12% ± 18.2%, *P* = 0.95 at 6 months; 4.82% ± 15.7% vs 8.74% ± 18.5%, *P* = 0.57 at 12 months; and 3.44% ± 21.3% vs 8.78% ± 16.0%, *P* = 0.48 at 24 months in the trabeculectomy vs the Ex-PRESS group, respectively). The 6A of corneal endothelial cells calculated by average of the five areas before surgery was 54.6% ± 9.02% and 56.3% ± 12.3% in the trabeculectomy and Ex-PRESS groups, respectively (*P* = 0.61). The postoperative percent reduction of 6A during the follow-up period also showed no significant differences between the two groups (2.09% ± 12.9% vs −2.69% ± 12.6%, *P* = 0.11 at 3 months; 3.42% ± 18.8% vs −3.01% ± 19.6%, *P* = 0.08 at 6 months; −1.81% ± 16.9% vs −4.01% ± 16.8%, *P* = 0.93 at 12 months; and −5.18% ± 20.9% vs −10.7% ± 24.9%, *P* = 0.38 at 24 months in the trabeculectomy vs Ex-PRESS group, respectively).

**Table 2:** The change rate of corneal endothelial cell density.

Time point	Trabeculectomy (%)	Ex-PRESS (%)	*P* value
3 months	1.1 ± 8.8 (n = 32)	−2.3 ± 7.9 (n = 32)	0.234
6 months	−1.4 ± 8.8 (n = 29)	−8.1 ± 11.0 (n = 32)	0.046*
12 months	−3.1 ± 8.0 (n = 25)	−10.1 ± 8.0 (n = 31)	0.004*
24 months	−2.2 ± 9.6 (n = 20)	−18.0 ± 29.1 (n = 28)	0.026*

Mann–Whitney nonparametric test with Bonferroni correction (data are mean values ± standard deviation). *Statistically significant (*P* < 0.05).

### Cataract progression and lens extraction after surgery

**Figure 1:**
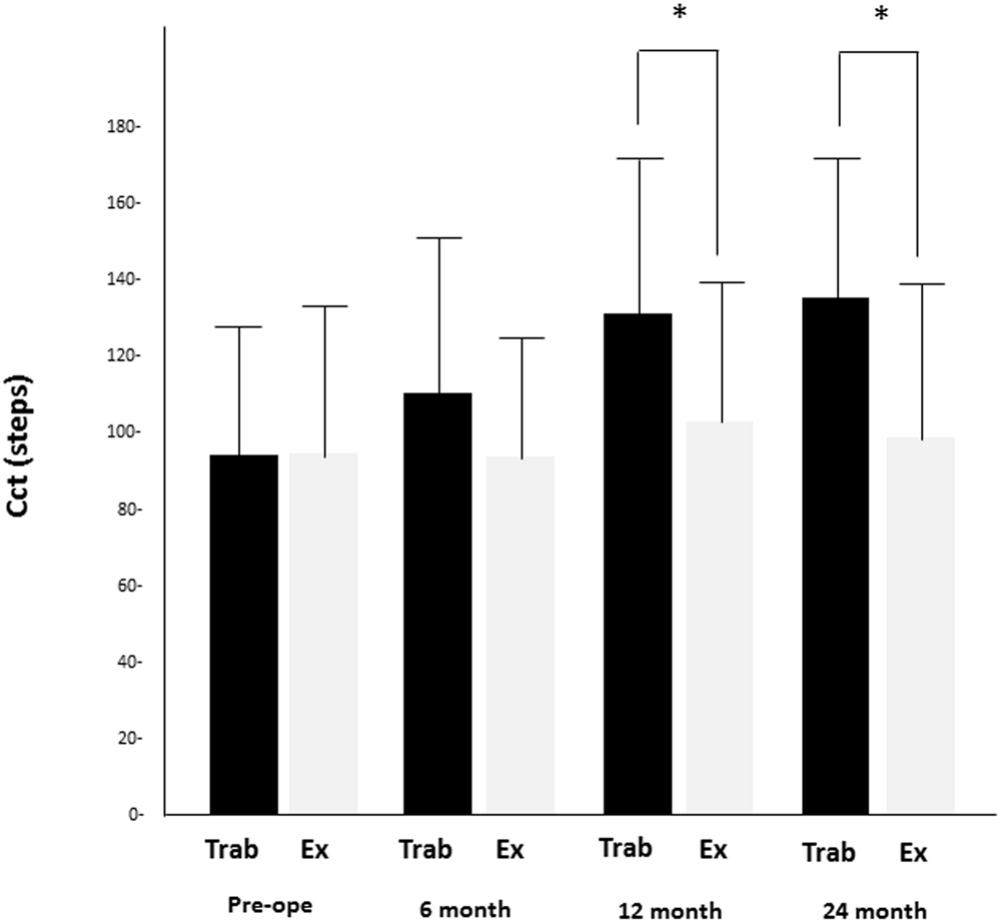
Comparison of light scattering intensity in lens nucleus after trabeculectomy and Ex-PRESS implantation. Trabeculectomy had a higher intensity of light scattering at 12 and 24 months (*P* = 0.02 at 12 months; *P* = 0.04 at 24 months). The data (mean ± standard deviation) were compared using a paired t-test with Bonferroni correction. Cct = the intensity of light scattering per pixel in each layer (Min; 0 steps, Max; 256 steps); Pre-ope = pre-operation; EX = the Ex-PRESS group; Trab = the trabeculectomy group.

**Figure 2:**
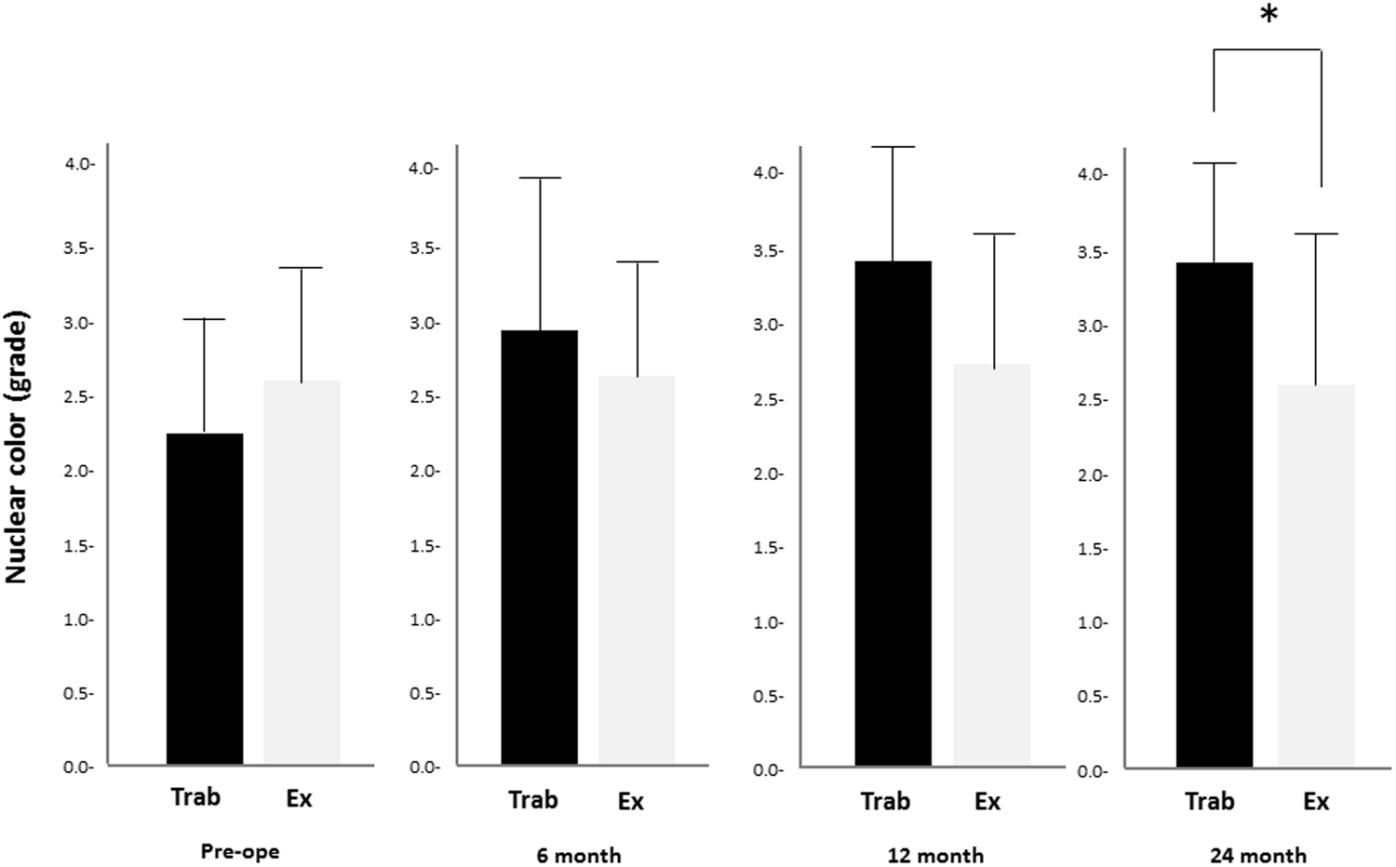
Nuclear colour changes based on Lens Opacification Classification System III after trabeculectomy or Ex-PRESS implantation. Trabeculectomy had a higher grade of nuclear colour at 24 months (*P* = 0.04). The data (mean ± standard deviation) were compared using the paired t-test with Bonferroni correction. Pre-ope = pre-operation; EX = the Ex-PRESS group; Trab = the trabeculectomy group.

There was no significant difference in the preoperative light scattering intensities of the lens nuclei between the two groups (94.2 ± 38.9 cct in the trabeculectomy group vs 94.1 ± 33.8 cct in the Ex-PRESS group; *P* = 0.89; Fig. 1). At 12 and 24 months after surgery, the trabeculectomy group had more light scattering intensity in the lens nuclei compared with the Ex-PRESS group (136.0 ± 43.0 cct vs 102.8 ± 36.7 cct, *P* = 0.02 at 12 months; 136.8 ± 42.5 cct vs 101.2 ± 41.9 cct, *P* = 0.04 at 24 months in the trabeculectomy vs the Ex-PRESS groups, respectively). Despite no significant preoperative difference in the nuclear colour grades between the two groups according to LOCS-III, the trabeculectomy group had a significantly more nuclear colour grade than the Ex-PRESS group did at 24 months (3.4 ± 0.7 and 2.7 ± 1.0 in the trabeculectomy and Ex-PRESS groups, respectively; *P* = 0.04; Fig. 2). Overall, 7 (21.9%) of the 32 eyes in the trabeculectomy group and 1 (3.1%) of the 32 eyes in the Ex-PRESS group underwent cataract surgery within 1 year (*P* = 0.017), and 12 (37.5%) of the 32 eyes in the trabeculectomy group and 4 (12.5%) of the 32 eyes in the Ex-PRESS group underwent cataract surgery within 2 years (*P* = 0.019).

As for the corneal ECD of the patients who had undergone cataract surgery during the follow-up period, the corneal ECD decreased significantly more in the Ex-PRESS group than in the trabeculectomy group followed by cataract surgery (−6.5% ± 5.5% in the trabeculectomy group vs −17.2% ± 10.1% in the Ex-PRESS group at 24 months; *P* = 0.045).

### Frequency of other postoperative complications

Other postoperative complications after trabeculectomy and Ex-PRESS implantation are reported in Table 3. Although no complications showed significant differences between the two groups, the total number of complications was significantly higher in the trabeculectomy group than in the Ex-PRESS group (*P* = 0.02).

**Table 3:** Other postoperative complications between 3 and 24 months.

Complication	Trabeculectomy (%)	Ex-PRESS (%)	*P* value
Bleb leak	2 (6.3%)	0 (0.0%)	0.08
Endophthalmitis	0 (0.0%)	0 (0.0%)	1.00
Shallow anterior chamber	2 (6.3%)	0 (0.0%)	0.08
Choroidal effusion	2 (6.3%)	1 (3.1%)	0.48
Hypotonic maculopathy	0 (0.0%)	0 (0.0%)	1.00
Total complications	6 (18.8%)	1 (3.1%)	0.02*

Chi-square test. *Statistically significant (*P* < 0.05).

### Secondary outcomes

#### Postoperative IOP

There were no significant differences in the IOPs between the trabeculectomy and Ex-PRESS groups at any postoperative visits (Table 4). The number of antiglaucoma medications was also not significantly different between the two groups at any postoperative visit. The number of patients who underwent reoperation due to insufficient IOP reduction was 3 (9.4%) in the trabeculectomy group and 2 (6.3%) in the Ex-PRESS group. The reoperation rate was not significantly different between the groups (*P* = 0.64).

**Table 4:** Intraocular pressure in the trabeculectomy and Ex-PRESS groups.

Time point	Trabeculectomy (mm Hg)	Ex-PRESS (mm Hg)	*P* value
Preoperatively	27.9 ± 10.7 (n = 32)	27.2 ± 8.5 (n = 32)	0.94
3 months	13.4 ± 3.8 (n = 32)	14.7 ± 6.0 (n = 32)	0.48
6 months	14.2 ± 4.4 (n = 31)	14.1 ± 3.8 (n = 32)	0.88
9 months	13.4 ± 3.9 (n = 30)	14.1 ± 4.0 (n = 31)	0.77
12 months	13.2 ± 3.7 (n = 29)	14.4 ± 3.6 (n = 30)	0.58
24 months	15.0 ± 4.4 (n = 25)	14.7 ± 2.2 (n = 28)	0.33

Mann–Whitney nonparametric test (data are mean values ± standard deviation).

### Postoperative visual acuity

The postoperative visual acuities, which were calculated as LogMAR values, showed no significant differences between the two groups (0.57 ± 0.72 and 0.51 ± 0.44; *P* = 0.41 at 6 months, 0.60 ± 0.65 and 0.58 ± 0.52; *P* = 0.69 at 12 months, 0.49 ± 0.60 and 0.51 ± 0.50; *P* = 0.38 at 24 months in the trabeculectomy and Ex-PRESS groups, respectively). The postoperative visual acuity of the patients who had not undergone cataract surgery during the follow-up period also showed no significant differences between the two groups (0.48 ± 0.61 and 0.51 ± 0.44; *P* = 0.16 at 6 months, 0.60 ± 0.68 and 0.60 ± 0.52; *P* = 0.60 at 12 months, 0.53 ± 0.67 and 0.55 ± 0.50; *P* = 0.32 at 24 months in the trabeculectomy and Ex-PRESS groups, respectively).

### Area dependency of corneal ECD reduction

The comparison of the decreasing rate for corneal ECD among the five photographed areas revealed a significant difference between the superior area versus the inferior area in the Ex-PRESS group. The superior area had a higher decreasing rate than the inferior area at 1 year and later (−13.8% ± 10.9% and −7.5% ± 10.6% at 1 year, *P* = 0.021; −17.6% ± 24.2% and −11.7% ± 24.0% at 2 years, *P* = 0.040 in the superior and inferior areas, respectively) (Table 5). In contrast, the trabeculectomy group had no area-dependent reduction of corneal ECD during the follow-up period.

**Table 5:** Comparison of change rate for corneal endothelial cell density between the superior and inferior areas in the Ex-PRESS group.

Time point	Central area (%)	Superior area (%)	vs	Inferior area (%)	*P* value
3 months	1.4 ± 10.2	−0.3 ± 9.1	vs	−2.1 ± 11.8	0.99
6 months	−10.7 ± 14.5	−10.1 ± 12.6	vs	−6.3 ± 15.4	0.30
12 months	−10.4 ± 9.01	−13.8 ± 10.9	vs	−7.5 ± 10.6	0.02*
24 months	−19.0 ± 30.2	−17.6 ± 24.2	vs	−11.7 ± 24.0	0.04*

Mann–Whitney nonparametric test with Bonferroni correction (data are mean values ± standard deviation). *Statistically significant (*P* < 0.05).

The change rate of central area had no significant differences compared to the other corneal areas.

## Discussion

The main aim of our present study was to compare postoperative complications following trabeculectomy and Ex-PRESS implantation over 2 years. There was significantly more reduction of corneal ECD after Ex-PRESS implantation than after trabeculectomy (*P* = 0.046 at 6 months; *P* = 0.004 at 1 year; *P* = 0.026 at 2 years). Subgroup analysis in the Ex-PRESS group showed a greater reduction of corneal ECD in the superior area than the inferior area (*P* = 0.021 at 1 year; *P* = 0.040 at 2 years). The postoperative cataract progression was more intense in the trabeculectomy group than in the Ex-PRESS group (*P* = 0.02 at 1 year; *P* = 0.04 at 2 years). More eyes in the trabeculectomy group were postoperatively followed by cataract surgery than eyes in the Ex-PRESS group (*P* = 0.017 at 1 year; *P* = 0.019 at 2 years). The number of other postoperative complications in 2 years was significantly higher in the trabeculectomy group than in the Ex-PRESS group (*P* = 0.02).

Despite the many reports^8,19,20^ comparing early postoperative complications between the two groups, few studies have reported a comparison of postoperative complications after more than 1 year. The XVT Study^9^ prospectively analysed 59 eyes with Ex-PRESS and 61 eyes with trabeculectomy for 2 years. Postoperative lens extraction was performed for 5.1% of the Ex-PRESS group and 11.5% of the trabeculectomy group, exhibiting no significant difference. Complications such as hyphema and over-filtration in the early postoperative period were significantly higher in the trabeculectomy group. Another randomised study compared the surgical outcomes of phacotrabeculectomy versus phaco-Ex-PRESS for 12 months after surgery^21^. Corneal ECD in the phaco-Ex-PRESS group was reduced by 37.4 ± 19.2% compared to a 23.2 ± 14.1% reduction in the phacotrabeculectomy group after 12 months follow-up. Another randomised trial followed patients for 3 years after either trabeculectomy (n = 31) or Ex-PRESS (n = 32)^22^. One patient in the Ex-PRESS group and five in the trabeculectomy group underwent cataract surgery within 3 years. Our study is unique because the comparison is focused on the complications after more than 1 year including quantification of corneal ECD reduction and cataract progression.

Corneal ECD reduction is frequently reported as a postoperative complication after filtering surgery^23–25^. Regarding Ex-PRESS implantation, two previous studies have compared corneal ECD between Ex-PRESS implantation and trabeculectomy. The corneal ECD reduction was compared among trabeculectomy (n = 22), Ex-PRESS implantation (n = 24), and Ahmed glaucoma valve implantation (n = 18) for 3 months after surgery^26^. Ex-PRESS implantation did not significantly reduce corneal ECD within 3 months while trabeculectomy and Ahmed glaucoma valve implantation resulted in a significant loss of corneal ECD. Compared with trabeculectomy, the Ex-PRESS filtration device is designed to reduce over-filtration during surgery and in the early postoperative period. The lower loss of corneal ECD with Ex-PRESS implantation in the previous study might reflect endothelial protection from the lack of over-filtration in the early postoperative period. Another randomised study compared corneal ECD between phaco-Ex-PRESS and phacotrabeculectomy for 1 year after surgery. The study showed significantly greater loss of corneal ECD in the phaco-Ex-PRESS group^21^. Although combined phacoemulsification might have enhanced corneal ECD loss after filtering surgery, the data is consistent with our present study. A single arm case series for eyes treated with Ex-PRESS implantation also showed a significant reduction in the corneal ECD^27^. The mechanism underlying the damage to the corneal endothelial cells following Ex-PRESS implantation remains unknown. Other glaucoma implants inserted into the anterior chamber also reduce corneal ECD during a long-term follow-up period^14,28–30^. A secondary outcome in our study was that the superior area had higher ECD reduction than the inferior area in the Ex-PRESS group whereas no area dependency was observed in the trabeculectomy group. The data suggest that the mechanism underlying the damage to the corneal epithelium following Ex-PRESS implantation might be different from that of corneal ECD loss after trabeculectomy. It is possible that the damage might be caused by foreign body reaction or immune reactions due to the stainless material used for the tube, the changed flow of the aqueous humour around the tip of the tube, or mechanical ablation between the corneal endothelium and the tube. Interestingly, among eyes that underwent cataract surgery^31,32^, the corneal ECD decreased more significantly in the eyes with Ex-PRESS implantation than in the eyes with trabeculectomy. Similar to the data, corneal decompensation frequently occurs after cataract surgery in eyes treated with tube shunt surgery. Taken together, cataract surgery might be associated with damage to corneal endothelial cells, which are compromised by the Ex-PRESS implantation.

Many previous studies have reported a higher progression of cataracts after trabeculectomy^33–37^. The reason for cataract progression after glaucoma surgery is not clearly understood. Eyes with trabeculectomy have a higher risk of cataract progression compared to those with non-penetrating deep sclerectomy^38^. A comparison of trabeculectomy and viscocanalostomy^39^ revealed a tendency for cataract progression in eyes treated with trabeculectomy (*P* = 0.066). The data suggest that hypotony in eyes with trabeculectomy is related to cataract progression. However, in our clinical trial, both postoperative IOPs and the frequency of hypotonic complications such as a shallow anterior chamber, choroidal detachment, and hypotonic maculopathy in the trabeculectomy group were comparable to those in the Ex-PRESS group in the early^8^ as well as the 2-year-postoperative periods. A recent cohort study for prophylactic laser peripheral iridotomy for eyes suspected of primary angle closure showed that eyes treated with prophylactic laser peripheral iridotomy were more frequently associated with cataract progression than eyes suspected of primary angle closure that had never undergone prophylactic laser peripheral iridotomy^40^. The results suggest that another opening site for aqueous humour in the iris facilitates cataract progression. Because iridectomy is not required for Ex-PRESS implantation, unlike trabeculectomy, Ex-PRESS implantation might result in a lower incidence of cataract progression compared to trabeculectomy.

The tendency for more frequent bleb leaks in the trabeculectomy group (P = 0.08) might contribute to the significantly higher frequency of postoperative complications (*P* = 0.02) in the group. A retrospective assessment of bleb morphologic features after Ex-PRESS implantation versus trabeculectomy revealed a more diffuse bleb area in the Ex-PRESS group^6^. Diffuse bleb formation might be related to a lower frequency of leaking bleb in the Ex-PRESS group.

The present study had some limitations. Firstly, we could not obtain significantly better postoperative visual acuity in the Ex-PRESS group despite the greater cataract progression in the trabeculectomy group. The discrepancy may be associated with the unrestricted inclusion criteria for preoperative visual acuity. Variation of preoperative data in each group results in a lower possibility of significant differences in the outcome. Eyes treated with cataract surgery postoperatively were excluded from the comparison of postoperative visual acuity between the two groups. This bias might have resulted in insufficient power to prove the superiority of the significant outcome. Second, the present data still do not offer long-term results for postoperative complications after 5 years or longer. Tube erosion or corneal decompensation is reported to be associated with Ex-PRESS implantation^13,41^. CPETS has been monitored for 5 years after surgery. Further follow-up periods might provide more information about postoperative complications for Ex-PRESS implantation.

In conclusion, 2 years of follow-up for the comparison of postoperative complications between trabeculectomy and Ex-PRESS implantation revealed that Ex-PRESS implantation is associated with a significantly greater reduction of corneal ECD, especially in the corneal area around the inserted tube, but less cataract progression than trabeculectomy. Ex-PRESS implantation is beneficial for preventing postoperative cataract progression. However, caution should be employed to prevent adverse effects against corneal endothelial cells. Therefore, Ex-PRESS implantation should not be considered for eyes with compromised corneal function.

## Electronic supplementary material

CONSORT 2010 Checklist

Protocol
