# Supplementary material for: Randomised Clinical Trial for Postoperative Complications after Ex-PRESS Implantation versus Trabeculectomy with 2-Year Follow-Up
Source: Sci Rep. 2018 Nov 1;8:16168. doi: 10.1038/s41598-018-34627-w (PMC6212395; doi:10.1038/s41598-018-34627-w)
Supplement: Supplementary file 2 — Protocol [file 41598_2018_34627_MOESM2_ESM.docx]

**Protocol**

**Patient Selection**

CPETS was approved by the institutional review board of Fukui University Hospital, Fukui, Japan. This study was registered with the University Hospital Medical Information Network Clinical Trials Registry of Japan (identifier University Hospital Medical Information Network 000008680; date of access and registration, August 15, 2012). The protocol adhered to the tenets of the Declaration of Helsinki. Written informed consent was obtained from all subjects. Patients were recruited between August 30, 2012 and February 13, 2015 at Fukui University Hospital. The enrolled patients were randomly assigned to receive either a trabeculectomy or an Ex-PRESS implantation with the following criteria: (1) Japanese patients with primary OAG or exfoliative glaucoma, (2) minimum age of 20 years, (3) no previous history of ocular surgery, and (4) an IOP ≥ 18 mmHg (despite maximum tolerated administration of anti-glaucoma medication). The exclusion criteria were as follows: (1) eyes that had not been treated with anti-glaucoma medication before surgery, (2) had a history of uveitis, or (3) were allergic to metals. For each patient, if both eyes satisfied the inclusion criteria, the eye with the higher IOP was selected for the study. The preoperative IOP was the average of the measurements taken within two months prior to the operation.

**Surgical Procedures**

All trabeculectomy and Ex-PRESS implantation procedures were performed using identical processes during the study period. A 5-mm conjunctival incision was created along the limbus to construct a fornix-based conjunctival flap, and a 4-mm wide half-layer scleral flap was formed. Mitomycin-C (0.4 mg/mL) was applied on and under the scleral flap and under the conjunctiva for 4 min, followed by irrigation with 200 mL of physiological saline. In the eyes that received a trabeculectomy, a deep block of limbal tissue was excised to create a fistula into the anterior chamber, and a peripheral iridectomy was performed. In the eyes that received an Ex-PRESS implantation, the tube was inserted into the anterior chamber after penetration with a 25-gauge needle. In both procedures, the scleral flap and conjunctiva were then sutured with 10-0 nylon. All patients received similar postoperative topical medications: 0.5% levofloxacin for 3 weeks and 0.1% betamethasone for 6 months after operation respectively. The laser suture lysis and bleb needling were completed within 1 month of surgery, depending on the postoperative IOP and formation of the bleb.

**Primary Outcome Measures**

The primary outcomes were postoperative complications, which included postoperative percent reduction of the corneal ECD, the coefficient of variance (CV) and the hexagonal cell appearance rate (6A) of corneal endothelial cell compared with preoperative corneal ECD, CV and 6A, nuclear cataract progression and the frequency of postoperative complications between 3 and 24 months. The change in corneal ECD, CV and 6A of corneal endothelial cell between preoperative and postoperative visits was quantified in 5 areas photographed (central, superior, inferior, nasal, and temporal areas) with a non-contact specular microscope (NSP-9900 Ⅱ, Konan, Nishinomiya, Japan) by 1 experienced examiner (S.A.). The patients who underwent cataract surgery or reoperation due to insufficient IOP reduction were excluded from the analysis of corneal ECD. Nuclear cataract progression was quantified using the Lens Opacification Classification System Ⅲ (LOCS-Ⅲ)^13^. The light scattering intensity of the lens was evaluated using an anterior eye segment analysis system (EAS-1000, Nidek, Gamagori, Japan). A camera unit was used to obtain a two-dimensional linear image of the cross-section of the anterior segment along 0° with a 200 W light source. The light scattering intensity (cct; the intensity of light scattering per pixel in each layer) of the images was analysed using Image J.

**Secondary Outcome Measures**

The secondary outcomes were the dependency of the corneal ECD reduction on the 5 photographed areas after surgery within each group, the comparisons of IOP and visual acuity between the 2 groups.

**Data Collection of Patient Characteristics**

The data regarding patient characteristics were gender, age, glaucoma type, and other preoperative ophthalmic data, including preoperative IOP, number of medications, corneal thickness, anterior chamber depth, axial length, best-corrected visual acuity, and visual field. Corneal thickness, anterior chamber depth, and axial length were measured with optical coherence interferometry (OA-1000; Tomey, Aichi, Japan). A logarithm of the reciprocal of the decimal BCVA was used to approximate the logarithm of the minimal angle of resolution (LogMAR). Visual field testing was performed using a static automated white-on-white threshold 24-2 perimetry program, SITA Standard (Model 750; Zeiss, Tokyo, Japan). If the eye had advanced visual field loss, a 10-2 perimetry program was used.

**Follow-up Visit**

CPETS was planned to examine postoperative complications for five years. Follow-up visits for the clinical trial after more than 1 month occurred at 3, 6, 12 and 24 months after the surgery. If the patients required additional treatment for complications, they were examined at additional follow-up visits.

**Sample Size**

The sample size was found to provide 80% power to prove (at a one-sided level of 0.05) the superiority of a significant outcome between the trabeculectomy and Ex-PRESS groups for an effect size of 0.7.

**Statistical Analysis**

JMP version 10.0 (SAS Institute, Inc. Cary, NC, USA) was used for statistical analysis. Univariate analysis was performed with Wilcoxon’s non-parametrical test, the paired t-test with Bonferroni correction, and the χ^2^ test. *P* values were considered statistically significant if less than 0.05.
